# Burden of Disease of Multiple Sclerosis in Korea

**DOI:** 10.4178/epih/e2012008

**Published:** 2012-11-30

**Authors:** Soo-Eun Chung, Hae-Kwan Cheong, Jae-Hyun Park, Ho Jin Kim

**Affiliations:** 1 Department of Social and Preventive Medicine, Sungkyunkwan University School of Medicine, Suwon, Korea.; 2 Center for Molecular Medicine, Samsung Biomedical Research Institute, Seoul, Korea.; 3 Department of Neurology, National Cancer Center, Goyang, Korea.

**Keywords:** Multiple sclerosis, Burden of disease, DISMOD II, Incidence, Prevalence, Quality of Life

## Abstract

**OBJECTIVES:**

Multiple sclerosis (MS) is an inflammatory demyelinating disease of the central nervous system. There are few reports on the burden of disease of MS, worldwide. The authors aim to estimate burden of disease and estimate the epidemiologic indexes of MS in Korea using available epidemiologic data.

**METHODS:**

Epidemiologic indexes were computed using DISMOD II software based on prevalence from nationwide survey, incidence estimated from extrapolation, mortality from National Statistics Office, and duration of disease from literature as input indexes. We calculated disability-adjusted life year (DALY) as a measure of premature mortality and disability, equivalent to years of healthy life lost due to a given condition.

**RESULTS:**

The incidence of MS in Korea was 0.1 per 100,000, higher in female than in male. The highest incidence was estimated in the age group between 35 and 44 years in male and age group between 25 and 29 years in female. Total burden of disease of MS was 1,394 DALY, comprised of 292 (21%) years of life lost and 1,101 (79%) years lived with disability. The mean age at onset of MS was 33 years old in men and 32 years old in female. Estimated duration of disease was 35 years in men and 40 years in female. Most of the DALY of MS occurred in the adult population between 25 and 54 years of age.

**CONCLUSION:**

Although MS is a rare disease in Korea, most of the DALY arises from young people, which results in a major financial burden on the patient, family, health system and society.

## INTRODUCTION

Multiple sclerosis (MS) is an chronic inflammatory disorder of central nervous system, manifesting as a widespread demyelination. It is characterized by its chronic course with intermittent relapses, finally ending up with a severe debilitation. In Korea, prevalence of MS is one of the lowest compared to the other countries in the same latitude. It is a significant public health problem, however, in that its prevalence peaks at the most productive age group, at the fourth decade [1-4]. High prevalence and subsequent life-long disability poses a heavy care burden, both direct and indirect, to household and community [3,5-7].

In Korea, MS is designated as one of the rare and intractable diseases, to which medical care cost is supported from governmental subsidy [8]. Estimation of disease burden of MS is required because it takes one of the highest priority diseases among the rare and intractable diseases. However, complex and delicate nature of the disease poses a challenge to the clinical diagnosis, which may lead to underestimation of the prevalence [7].

Prevalence of MS has wide variation depending on regions, highest prevalence is reported in the regions between 45° and 65° latitude [9]. Adjusted prevalence of MS in the United Kingdom (average latitude is 54.6 N) is 21 per 100,000, but 4 per 100,000 in northern Latin America (average latitude of 20.7 S) [2,9,10]. In Korea, Kim et al. [11] reported 3.5 per 100,000 through national survey in 2006, which is one of the lowest in the world, even compared to that of the 3.9 in 100,000 in Morioka, Japan, located at the similar latitude in the neighboring country [12,13].

There is a limitation in the use of common epidemiological indexes [6,14]. Such as incidence or prevalence in reflecting quality of life, loss due to disability, or health status after recovery [15]. For this end, indicators of more comprehensive health outcome such as disability-adjusted life years (DALY), disability-free life expectancy and quality-adjusted life year have been developed [16,17]. Among these indicators, DALY, developed by World Health Organization (WHO) enables comparison of disease burden between countries and it is widely used for its clarity and simplicity in reflecting mortality and disability in a single index [18].

WHO reported the DALY of major neurologic diseases as 11,078 person-years for Alzheimer's disease, 7,308 person-years for seizure disorders, 1,617 person-years for Parkinson disease, which takes 0.75%, 0.50%, and 0.11% of total DALY, respectively [19]. DALY for MS was 0.10%, similar to that of the Parkinson disease. Compared to neurodegenerative diseases, MS is a disease of younger population, with higher socioeconomic and public health implication. In other words, years with disability (YLD) being higher than years of life lost (YLL) in MS, it takes a higher care burden leading to economic burden of the household [7].

WHO study reported that there is more than 2.5 million MS patients worldwide, taking 6.29% of total neurologic diseases, which is expected to increase 6.39% in 2015 and 6.77% in 2030 [19]. In Canadian study, younger generation takes half of total incidence and death of MS and most of the medical expenditure was raised from treatment and care of the disease and disability, representing higher burden from disability compared to that from death [20].

In Korea, there is no estimation of disease burden of MS and WHO estimation of burden of disease may not reflect the country-specific epidemiologic indexes [8], thus having a limitation in applying to a specific country. Therefore, the authors intended to estimate the burden of disease for MS for age and gender groups, using up to date epidemiologic indexes, and to estimate other basic epidemiologic indexes for the MS.

## MATERIALS AND METHODS

### Data source

In order to calculate the burden of disease suggested by WHO, epidemiological indexes such as prevalence, incidence, case fatality rate and remission rate are needed [18]. For prevalence, this study uses the rates by five-year age and gender groups nationwide included in the study by Kim et al. [11], which was estimated based on examined health insurance data and hospital survey on medical records.

The study is the only report of prevalence in nationwide level in Korea, and presents prevalence by age and gender for all age groups. Because there is no domestic report on incidence, data from overseas were used to estimate incidence for each five-year age group. For children and youth between 0 and 14 years of age, incidence of Japan (Fukuoka 34° of latitude), whose prevalence is most similar to that of Korea, was used [21]. For over 15 years of age for whom Japan's data were not sufficient, prevalence was estimated by using a report from Italy (Ferrara 44° of latitude). This report presents incidence and prevalence for all age groups. The study site Ferrara is located at a lower degree of latitude than other European regions but at similar latitude to Japan (26-46° of latitude). In addition, the region showed the most similar prevalence, relatively, to Asia [22].

**Figure 1:**
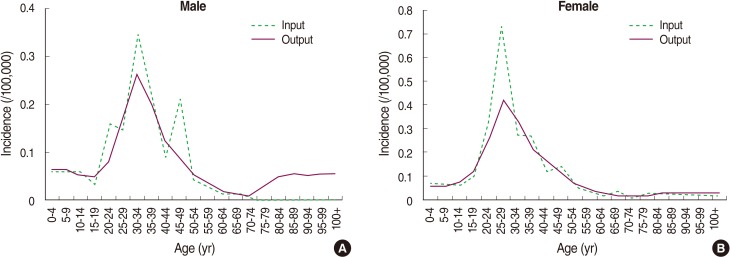
Estimated input and output data model of incidence by age and sex in DISMOD II, males (A) and females (B).

Estimation of incidence by age was premised on the fact that prevalence and incidence are proportional to each other if the duration of disease is fixed, in order to calculate the proportion of prevalence of Italy and Korea, based on which incidence was also calculated. In this study, incidence of MS patients was missing for patients younger than 10 and older than 70, and thus gender and age were set as independent variables based on numerical values estimated from 10 to 69 years, and then a simple regression analysis was performed to calculate estimated incidence by age and gender (Figure 1). As for case fatality rate, because the data of death report of MS by National Statistics Office in Korea were assessed to be inappropriate due to underreporting, the mortality of Austria [23], which offers age- and gender-specific data, was used to be substituted with the following equation along with the calculated estimates of the MS prevalence [24]:

Case fatality=mortality/prevalence

Remission rate refers to a fraction of individuals with a disease recovering to a normal state, and '0' was applied as suggested in the Global Burden of Disease (GBD) project of WHO in consideration of the irreversible nature of MS [18]. Indexes for the calculation of burden of disease, such as disease duration and age of onset, which have not been reported domestically, were calculated by using DISMOD II software. DISMOD II is a software developed by WHO to estimate epidemiological indexes of disease based on the mathematical relationship between epidemiological indexes [25]. For input indexes, those with the most approximate values after calculation through substitution were applied to raise validity of the output.

At least three of the indexes other than age- and gender-specific population, among prevalence, incidence, mortality, disease duration and age at onset, were needed to estimate the indexes [25,26]. In this study, prevalence, incidence, case fatality and remission rates, obtained as described, were selected as input indexes and allocated them by five-year age group and gender to be used for the input indexes of the software. For disability weight, 0.68 was used, which was calculated based on the Dutch Disability Weights Group's protocol introduced in the GBD study [18,24].

### Burden of disease

For comprehensive evaluation of death and disease on health effects, the following formula was used for the calculation of DALY [19,27-29]:

D isability-adjusted life year (DALY)=years of life lost (YLL)+years lived with disability (YLD)

YLL[r,K]= KCe^ra^/(r+β)^2^{e^-(r+β)(L+a)^[-(r+β)(L+a)-1]-e^-(r+β)a^[-(r+β)a-1]}+[(1-K)/r] (1-e^-rL^)

YLD[r,K]= D{KCe^ra^/(r+β)^2^{e^-(r+β)(Ld+a^)[-(r+β)(Ld+as)-1]-e^-(r+β)a^[-(r+β)a-1]}+[(1-K)/r](1-e^-rL^)

L, average expected years remaining; r, discount rate (0.03); K, age weight modulation factor (1); a, age at death; β, age weight basis (0.04); L, average expected years remaining; D, disability weight (0.690) [17-19].

In this study, values for incidence, prevalence, case fatality, mortality, disease duration and age at onset were entered into the YLD expression, and fatality rate was added to the YLL formula for calculation. The DALY equation was applied in the same way as in the GBD study, and 0.04 (K=1) was applied as age weight β, while the discount rate was calculated to be 3%. Through this, the total DALY of MS was calculated with a unit of 100,000 population, and YLL and YLD values by gender and age, and the percentages of each value was also obtained.

## RESULTS

**Figure 2:**
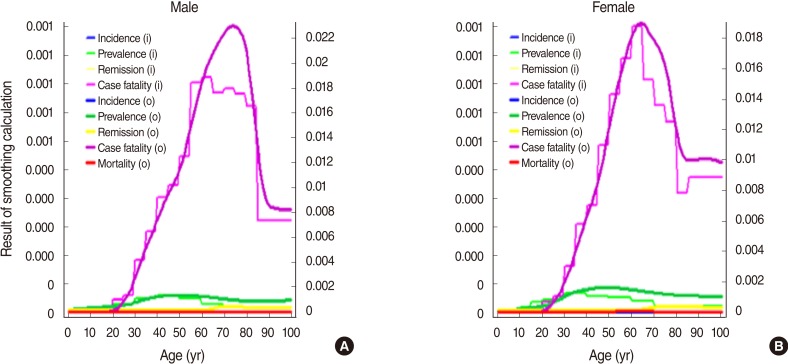
Input (i) and output (o) variables in DISMOD II modeling. Calculated from prevalence, incidence, case fatality and remission in multiple sclerosis using DISMOD II, males (A) and females (B). Calculation option: piecewise linear interpolation, moving average, cubic spline. (A) males and (B) females.

The epidemiological indexes obtained by entering prevalence, estimated incidence, estimated case fatality and remission rates into DISMOD II as input variables were compared with the estimated prevalence and incidence. We found that both inputs and outputs had almost the same distribution, indicating their sufficient validity (Figure 2). The resulting prevalence of epidemiological indexes was between 2.0 and 5.8 per 100,000 in the 20 to 59 age group in men and between 2.4 and 8.4 in women per 100,000. For five-year age groups from 20 to 59, men had the highest rate (5.78/100,000/year) in the 50-54 age group, which began to decrease from over 55 years of age, whereas women had the highest rate (8.36/100,000/year) in the 45-49 age group, which began to decrease from over 50. The result of incidence output in the five-year age group between 20 and 59 by gender, men had the highest rate (0.26/100,000/year) in the 30-34 age group whereas women had the highest rate (0.42/100,000/year) in the 25-29 age group, indicating that women had a higherincidence than men (Table 1).

DALY of MS in the Korean population in 2008 was 2.9 person-years per 100,000 and women had about 44% higher rate than men. DALY of MS tended to increase from 22 to 44, peaked at 25-29, then declining from over 50 (Table 2). YLL was 117 for men and 176 for women, whereas YLD was 435 for men and 666 for women, with a total of 1,101 years. Compared to men, women had a higher in the total YLL as well as YLD (Table 2).

**Figure 3:**
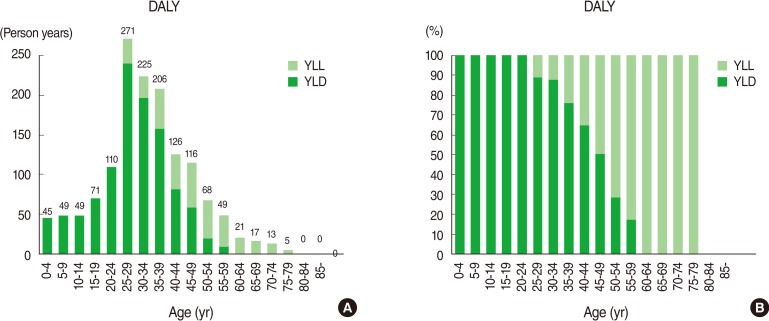
Years of life lost (YLL), years lived with disability (YLD) and disability-adjusted life year (DALY)s due to multiple sclerosis by age in Korea. (A) Age-specific composition of YLL and YLD of MS by age group. (B) Proportion of YLL and YLD of MS by age group. DALY=YLL+YLD [14].

It is estimated that the average age at onset for MS patients in Korea is 33.2 for men and 32.2 for women, and the case fatality rate was 933.5/100,000 for men and 942.5/100,000 for women. The duration of disease or survival was 35 years for men and 40 years for women, longer in women. The disease burden of MS between 20 and 54 of age was 1,124 person-years, which is 78% of the entire DALY of 1,444 person-years (Figure 3).

## DISCUSSION

DALY of MS in the entire Korean population in 2008 was estimated to be 2.9 person-years per 100,000 persons, and total DALY was higher in women than in men, and was the highest in the 20-30 age group. YLD accounted for 79% of the total, indicating that most of the burden of disease comes from the loss due to disability from the disease.

MS occurs mainly among younger people, and has high incidence among adults of productive age from 20s to 50s. Thus, DALY of age group between 20 and 54 years of age took 78% of the entire DALY. High prevalence among the younger age groups is not directly linked to death, but once diagnosed, having MS means that patients suffer from it for the rest of their lives, which add socioeconomic cost burden on corresponding population group [18,30,31].

Health indexes using standardized prevalence and incidence cannot be easily readjusted for age-weighted and health discount rate over time. Moreover, domestic research on quality of life is limited to certain diseases with high prevalence, and no research has been performed on rare diseases. There is no clear idea about the burden of disease study at the national level required for medical policies, either [15].

WHO, in GBD study, reported that the DALY for MS was 23.4 person-years per 100,000 persons [14], which accounts for 0.10% of all diseases, a much higher level compared to this study. One reason can be that the WHO study included countries with notably higher prevalence such as European countries and Canada as MS has drastically varying prevalence depending on the latitude [10]. A region at a higher degree of latitude tends to have a higher prevalence. This difference is an environmental risk factor of MS in addition to the genetic factor, and it was discovered that differences resulting from local environments and race based on the latitude of locality and migration have partial effect, but it is still unclear in terms of etiology, leaving room for controversy [4].

However, compared to the low prevalence and incidence among Asian races, the survival has no significant correlation with latitude of other regions including Europe, and the fact that it is 25-48 years old globally with no great difference [10,11,18,29,30] means that while there clearly is an environmental difference when it comes to the occurrence of the disease, pathogenesis characteristics such as disease's progress and age had little difference.

The burden of disease of MS estimated in this study cannot be compared with other rare diseases due to lack of research data on them, but when compared with individual diseases studied in Korea such as chronic illnesses, it was at a very low level [17]. In terms of demographic characteristics, DALY of 20-54 years old accounted for the majority of MS, but DALY of all neurological disorders took up 63.3%, or the majority, in the age group of over 55 in the aged population, showing a different trend from MS [11,31,32]. This confirms that while MS has low incidence compared to other neurological disorders, it can bring about a huge burden as it occurs at an early age. In the case of Japan whose epidemiological characteristics have the greatest similarity to those of Korea, its incidence is slightly higher than Korea's, but its prevalence is on the steady rise [12,33]. Given that Korea's domestic circumstances are similar, one can anticipate that MS will take up a higher portion of the entire burden of disease.

This study aimed to measure disease burden of MS reflecting Korea's situations, and to that end, representative data on prevalence from hospitals and national health insurance were chosen for use among major epidemiological indexes [11]. While the data on prevalence were appropriate for use in this study as they divided patients across Korea into separate age groups, the data to calculate prevalence were insufficient due to limitations in terms of data collection. To complement this shortcoming and give sufficient consideration to the domestic circumstances, a country in Asia whose racial and regional characteristics are the closest to those of Korea, in order to calculate estimated incidence. Of the collected literature, data of Japan as a neighboring country were chosen as the most suitable candidate that satisfied the aforementioned conditions.

However, for missing incidence rates among a few specific age groups, the study team was unable to find data among Asian countries with similar racial and geographical characteristics that could fill in the gap for the Japan's missing data. As such, the study team chose the data from Ferrara, Italy, which is located at a relatively approximate degree of latitude of European countries through literature survey that gave maximum consideration to regional characteristics [23].

The study on MS of the Ferrara region offers accurate reports of the local population and includes all incidence and prevalence by gender and age group with high data reliability. Hence, the data were considered to provide a sufficient basis to supplement the missing data. Values calculated by using such data were applied to a regression equation in consideration of the relations between domestic incidence and prevalence, as well as domestic circumstances, to estimate the final incidence in Korea.

When this was cross-examined with the outcome from DISMOD II, the overall incidence level and incidence distribution by age and gender were found similar to the input and output of DISMOD II, proving the estimated data's validity. The calculated duration of disease was 35 years for men and 40 years for women in consideration of mean age. While there is no domestic report on the duration of disease, it was 36 years for men and 43 years for women among MS patients in Norway. More recently, a Canadian study reported it to be 32.5-49.2 years [34], confirming its validity [30,35,36].

This study is limited in that the available data did not cover the same race in the same region, and as such they could not reflect domestic circumstances accurately. However in reality, domestic data either lack incidence, survival time and fatality rate classified into different age groups or came with restrictive conditions, which made it impossible to utilize. To address this shortage, approximate data were estimated by combining research outcomes home and abroad by considering various factors and environment in Korea. Moreover, the disability weight of other diseases studied domestically is somewhat different from the WHO standards, which indicated that criteria of MS patients have different domestic and foreign conditions. Thus it was considered impossible to reflect all conditions entirely. Even when the WHO standards, which are widely known as the world standards, were applied, they still have some discrepancies with the actual domestic environment.

Like this, estimation of prevalence of rare diseases was limited. However, when one or more accompanying disease is associated, it restricts estimation of all other diseases' burden. The fact that evaluation is impossible without calculation of correlations between different diseases is a common limitation found in any study on the burden of disease [31,32]. In the case of MS, the estimated prevalence rate was the highest among a population over 90 years of age, but this seems to be because the total population of the most aged group is drastically smaller. Because MS incidence is relatively higher among younger age groups, it often progresses without any relevance to old-age diseases and associated chronic diseases. Thus, the purpose is to calculate the burden of individual diseases.

All other studies aiming to calculate disease burden using DALY had limitations as they failed to reflect quality of life with or without disability, respectively, due to the use of a uniform method, and divide patients by age and gender only, thereby failing to acknowledge differences resulting from income levels and environment [14,24]. The prevalence data used in this study, while falling short of being a census or a large-scale cohort study, are rare epidemiological data that cover cases of a rare disease nationwide and are subdivided by age and gender. Thus, the data serve not only as a sufficient basis for a national index but also a starting point of research on burden of other diseases, as it is the only nationwide study on the burden of a rare disease. The incidence estimated in this study and the duration of disease seem to be at similar levels with overseas data with a different prevalence.

In addition, MS was found to have a slightly lower incidence rate at an old age compared to Parkinson's disease whose incidence increases with aging. From the perspective of social public health, longer years lived with disability require continued management and alternatives at the national level, and recurring and aggravating nature of the disease places disease burden on the community, subsequently leading to a huge loss [8]. Moreover, considering the reality that hospitals specializing in MS do not have fully unified criteria for diagnosis and conditions for insurance calculation, epidemiological indexes that reflect domestic circumstances are a must to develop accurate index, if for nothing else.

This study is the first of its kind in Korea that utilized reliable domestic data to calculate the burden of disease of MS. This attempt is highly meaningful because nationwide reports on the burden of MS are rarely found around the world. Also, it succeeded in estimating objective disease burden that can be compared with other diseases, thereby offering socioeconomic benefits. Moreover, it can serve as a basis of epidemiological data for an individual disease that can fill in the gap of research on rare and incurable diseases whose research has been lagging in Korea. The study will contribute to establishing economic effectiveness and priorities in implementing health policies. If more accurate indexes that consider sociodemographic and local characteristics can be obtained by applying medical cost to be calculated in the future and demographic-specific data, they can have great use as a basis for national health economy and medical policy-planning. This study should be complemented by further research that approaches improvement of quality of life of patients suffering from rare and incurable diseases in a realistic sense by showing the changing epidemiological characteristics at home and through accurate measurement of incidence and fatality of a disease to ensure accurate understanding of the level of disease burden.

## Figures and Tables

**Table 1:**
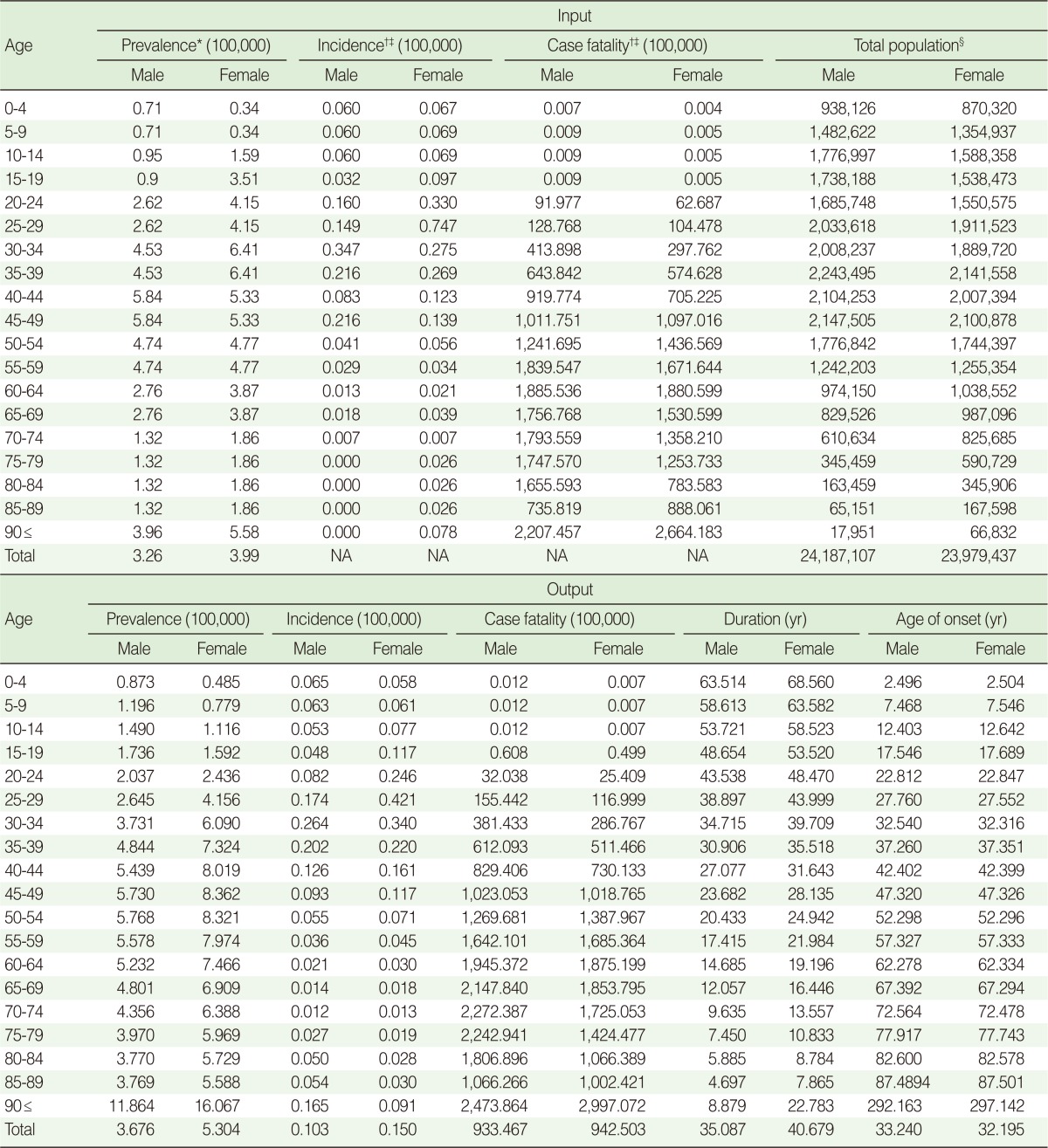
Input and output variables in DISMOD II modeling for multiple sclerosis in Korea

NA=not available. ^*^From Kim NH, et al., Prevalence of multiple sclerosis in Korea. Neurology 2010;75:1432-1438 [11]. ^†^From Torisu H, et al., Clinical study of childhood acute disseminated encephalomyelitis, multiple sclerosis, and acute transverse myelitis in Fukuoka Prefecture, Japan. Brain Dev 2010;32:454-462 [21]. ^‡^From Granieri E, et al., Multiple sclerosis in the province of Ferrara: evidence for an increasing trend. J Neurol 2007;254:1642-1648 [22]. ^§^From Statistics Korea. Population of Korea. (Internet) [24].

**Table 2:**
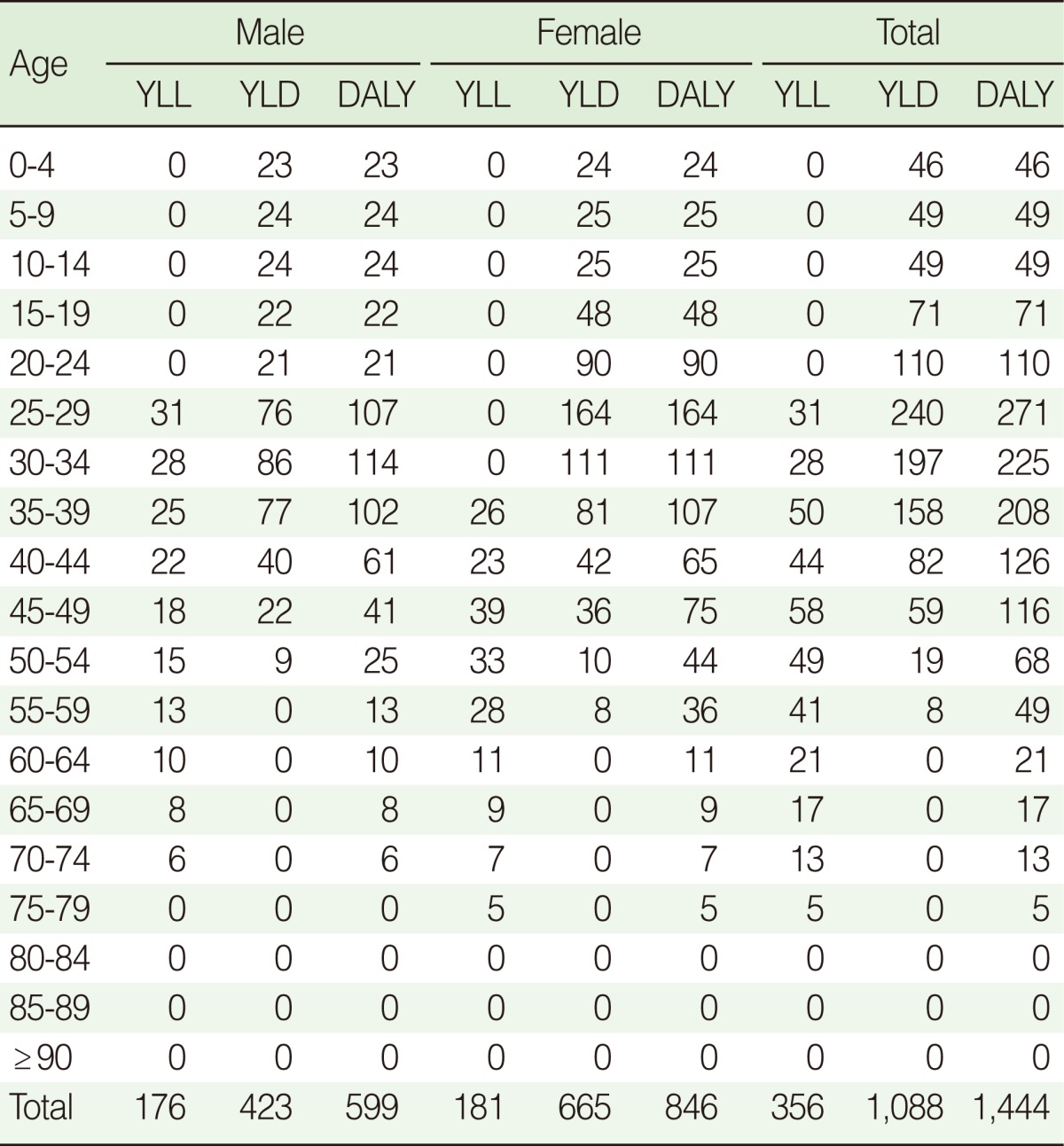
Years of life lost (YLL), years lived with disability (YLD) and disability-adjusted life year (DALY)s due to multiple sclerosis in Korea

Values are presented as person years.
